# The natural history of glycogen storage disease type Ib in England: A multisite survey

**DOI:** 10.1002/jmd2.12200

**Published:** 2021-01-24

**Authors:** Rebecca Halligan, Fiona J. White, Bernd Schwahn, Karolina M. Stepien, Nazreen Kamarus Jaman, Mel McSweeney, Steve Kitchen, Joanna Gribben, Charlotte Dawson, Katherine Lewis, David Cregeen, Helen Mundy, Saikat Santra

**Affiliations:** ^1^ Inherited Metabolic Disorders Birmingham Children's Hospital Birmingham UK; ^2^ Inherited Metabolic Diseases Evelina London Children's Hospital London UK; ^3^ Willink Unit Manchester Childen's Hospital Manchester UK; ^4^ Adult Inherited Metabolic Medicine Salford Royal Hospital NHS Foundation Trust Salford UK; ^5^ Metabolic Medicine Department Great Ormond Street Hospital London UK; ^6^ Inherited Metabolic Diseases Queen Elizabeth Hospital Birmingham UK; ^7^ Inherited Metabolic Diseases Guy's and St Thomas' NHS Foundation Trust London UK

**Keywords:** 1,5‐anhydroglucitol‐6‐phosphate, genotype, glycogen storage disease type Ib, liver transplantation, neutropaenia, neutrophil dysfunction

## Abstract

Glycogen storage disease type Ib (GSDIb) is characterized by hepatomegaly and fasting hypoglycaemia as well as neutropaenia and recurrent infections. We conducted a retrospective observational study on a cohort of patients with GSDIb across England. A total of 35 patients, with a median age of 9.1 years (range 1‐39 years), were included in the study. We examined the genotype and phenotype of all patients and reported 14 novel alleles. The phenotype of GSDIb in England involves a short fasting tolerance that extends into adulthood and a high prevalence of gastrointestinal symptoms. Growth is difficult to manage and neutropaenia and recurrent infections persist throughout life. Liver transplantation was performed in nine patients, which normalized fasting tolerance but did not correct neutropaenia. This is the first natural history study on the cohort of GSDIb patients in England.

## Graphical abstract

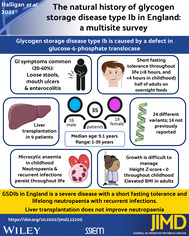

**Synopsis**

GSDIb in England is a severe disease with a short fasting tolerance and lifelong neutropaenia with recurrent infections.

## 1 INTRODUCTION

Glycogen storage disease type Ib (GSDIb) is caused by a defect in glucose‐6‐phosphate translocase (G6PT), which is an enzyme involved in the transportation of glucose‐6‐phosphate from the cytoplasm to the endoplasmic reticulum (ER).^1^ Once inside the ER, glucose‐6‐phosphate is cleaved by the enzyme glucose‐6‐phosphatase to release free glucose. GSDIb therefore results in a loss of the final step of both glycogenolysis and gluconeogenesis, leading to hepatomegaly and a short fasting tolerance with severe hypoglycaemia. GSDIb is also associated with neutropaenia and neutrophil dysfunction causing recurrent infections and inflammatory bowel disease (IBD).^2^ This has recently been attributed to an increase in 1,5‐anhydroglucitol‐6‐phosphate (1,5AG6P), which has a detrimental effect on glycolysis in neutrophils.^3^

The incidence of GSD type I (GSDI) is around 1 in 100  000, although this is higher in the Ashkenazi Jewish population, and GSDIa is much more frequently diagnosed than GSDIb. GSDIb is a pan‐ethnic autosomal recessive disease caused by pathogenic variants in *SLC37A4* (OMIM#232220). Although no clear genotype‐phenotype correlations have been made, there are several common mutations found among different ethnic groups. This includes the c.352 T > C (p.Trp118Arg) mutation described in over 50% of Japanese GSDIb patients, and the c.1015G > T (p. Gly339Cys) and c.1042_1043delCT (p.Leu348Valfs) mutations reported in European and German populations.^1^, ^4^

The management of GSDIb should allow for good growth and avoidance of secondary metabolic disturbances. Renal complications include nephromegaly and hypertension as well as both proximal and distal renal tubular dysfunction.^5^ Anemia is common and is felt to be linked to bowel inflammation and poor absorption of iron.^2^ Monitoring of long‐term complications such as hepatic adenomas and osteopaenic bone disease is also recommended.^6^ Due to the short fasting tolerance, the dietary management of GSDIb can be challenging and will usually involve overnight enteral feeds and frequent daytime feeds initially. As glucose requirements decrease with age, fasting tolerance is expected to increase, which is aided by the introduction of uncooked cornstarch (UCCS). However, patients with GSDIb often have difficulty tolerating UCCS due to bowel inflammation and resultant loose stools.^2^

Traditionally, neutropaenia has been managed with regular injections of granulocyte colony‐stimulating factor (GCSF); however, there is now growing evidence of the beneficial effect of renal sodium‐glucose co‐transporter‐2 (SGLT2) inhibitors in GSDIb through the reduction in 1,5AG6P levels.^3^, ^7^

There is variation in the management of GSDIb across both England and the world, with some centers advocating for liver transplantation in extreme cases.^2^, ^6^ The aim of this study was to describe the cohort of patients in England with GSDIb.

## 2 METHODS

Formal research ethics committee approval and individual patient consents were not required for this retrospective observational study where no patient identifiable data is presented. The study was supported by the Association for Glycogen Storage Disease UK (AGSD‐UK).

A survey of practice was distributed to all pediatric and adult metabolic centers in England. For pediatric patients, de‐identified patient data were collected on genotype and a range of phenotypical features in 2 yearly intervals up to the age of 16 years. De‐identified data on adult patients were collected in 5 yearly intervals.

## 3 RESULTS

De‐identified patient data were collected from 35 patients across England, with a total of 19 female and 16 male patients. Data were collected from 28 pediatric and 7 adult patients with the median age of patients included in the study being 9.1 years (range 1‐39 years). The median age for diagnosis of GSDIb was 4 months, with several patients not diagnosed until their third year of life and two patients diagnosed incidentally in adulthood. Nearly half of the cohort was of Pakistani ethnicity (46%) with the remainder identifying as South‐Asian (17%), White‐British (20%), Middle Eastern (3%) and “White‐Other” ethnicity (17%), which included heritage from South Africa and across Europe.

### 3.1 Biochemistry and hematology

Monitoring of urate levels throughout life showed that these were stable and easy to maintain within the normal reference range of 120 to 430 μmol/L, although they tended to be around the upper limit of normal in adolescence and adulthood. Almost all adult patients were treated with allopurinol. Triglyceride levels fell rapidly following diagnosis and tended to remain slightly above the upper limit of the reference range across life (reference range of 0.6‐1.9 mmol/L).

Patients had microcytic anemia across childhood, which was defined as a hemoglobin level less than 110 g/L under the age of 6 years or less than 115 g/L in those older than 6, with a mean cell volume (MCV) of less than 80 fL. This tended to improve slightly in adulthood. Neutropaenia was defined as an absolute neutrophil count below 1.5 × 10^9^/L in those younger than 12 and below 2 × 10^9^/L in older children and adults. Neutropaenia developed toward the end of the first year of life in most cases, and the median absolute neutrophil count remained below the lower limit of the reference range across childhood. Data on neutrophil count were incomplete for the adult patients. At least 50% of patients in all age points in data collection were treated with GCSF, with dosage varying between individuals but most commonly given three or five times per week. In all age points, 10% of patients were treated with vitamin E.

### 3.2 Growth

**FIGURE 1:**
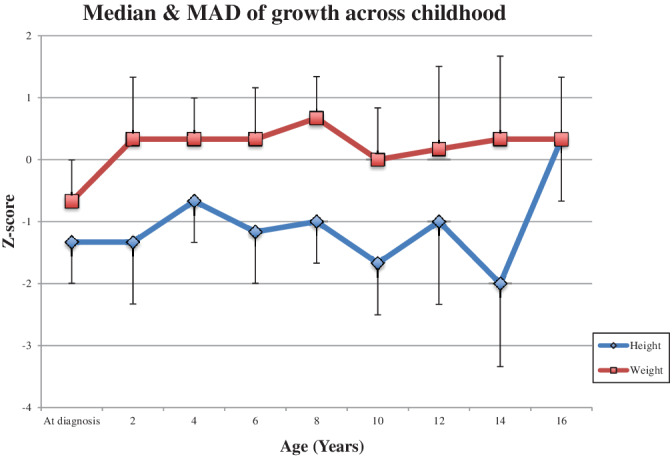
Median and median absolute deviation (MAD) of Z‐scores for height and weight across childhood in a cohort of GSD1b patients

Figure 1 shows the median and median absolute deviation (MAD) age‐and‐sex‐adjusted Z‐scores for height and weight across childhood in comparison to UK‐WHO growth charts, with the 50th centile representing the median for the population and each centile line above and below the 50th centile representing two‐third of a SD. No statistically significant difference was seen given the large variation in numbers. The median body mass index (BMI) for the eight adult patients was 26 kg/m^2^. One patient had a diagnosis of growth hormone deficiency and had previously been treated with growth hormone, which had not improved her final height.

### 3.3 Fasting tolerance

**FIGURE 2:**
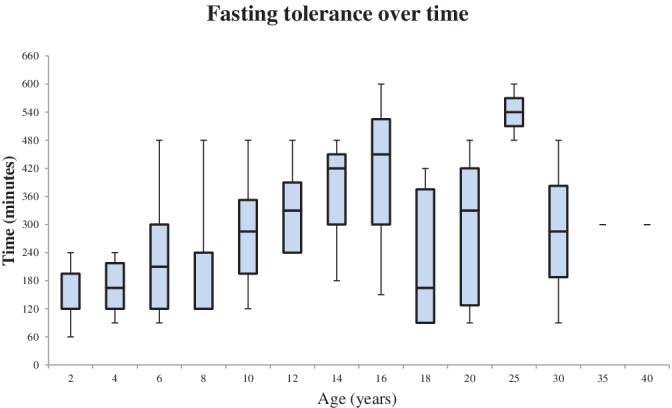
Median and interquartile range for fasting tolerance across life

Figure 2 shows the median fasting tolerance and interquartile ranges across all age groups, excluding patients post liver transplantation, in all of whom fasting tolerance had normalized. Fasting tolerance tended to be less than 8 hours throughout life, with early childhood fasting tolerance usually less than 4 hours. Half of the adult patients remained on overnight enteral feeds. The exception to this is the 25‐year age group where data for only two siblings were available, both of whom were diagnosed in adulthood.

### 3.4 Renal

Monitoring of protein/creatinine or albumin/creatinine ratios was inconsistently performed. Protein/creatinine ratios were more commonly performed and were either in the normal or upper limit of normal ranges across life (reference range of <20 mg/mmol). However, albumin/creatinine ratios performed in several adult patients showed a median level that was nearly double the upper limit of the reference range (reference range of <3 mg/mmol) and three adult patients were on treatment with angiotensin‐converting enzyme inhibitors due to proteinuria. Two patients developed nephrolithiasis one of which also developed nephrocalcinosis and proximal tubulopathy, leading to end stage renal failure requiring renal replacement therapy and transplant.

### 3.5 Gastrointestinal

The prevalence of gastrointestinal symptoms across all age groups ranged between 20% and 60%. Loose stools were the most commonly documented gastrointestinal complaint, seen in 30% of patients at age 2, followed by mouth ulcers, which were reported in 8% of patients when aged 6. Four patients were reported to have perianal abscesses. Enterocolitis was frequently described although only one pediatric patient had a formal diagnosis of IBD made by a pediatric gastroenterologist on endoscopy. Three adult patients were reported to have IBD although it is unclear how the diagnosis was made. No patients were found to have hepatic adenoma or carcinoma detected on routine monitoring using ultrasonography. Magnetic resonance imaging of the liver was performed infrequently, with no tumors identified.

Five patients had gastrostomy insertion with all reporting recurrent infections around the site and two patients requiring removal of the device.

### 3.6 Bone health

Data on bone health were only collected for adult patients and were infrequently performed. Only three patients had recent imaging using dual‐energy absorptiometry scans (DEXA), two of which showed mildly reduced bone mineral density, which was defined as a Z score between −1 and −2.5 when compared to age‐and‐sex‐matched controls.

### 3.7 Endocrine

One patient had a diagnosis of polycystic ovarian syndrome, which was investigated by ultrasonography. One female patient had a diagnosis of hypogonadotrophic hypogonadism and was under the care of the endocrine team. Another patient suffered early pregnancy loss and there were no other reported pregnancies. Data on male fertility were not collected.

### 3.8 Genotype

Table 1 shows the genotype seen in our cohort of patients including the allele frequency and American College of Medical Genetics and Genomics (ACMG) category.^8^ The diagnosis of GSDIb was made by molecular analysis of *SLC37A4* in almost all patients; however, reports were not available for three patients at the time of data collection. The diagnosis was made clinically and histologically on liver biopsy in one patient. Twenty‐four different variants were seen including 14 that have not been previously reported. The human genomic variant search engine, VarSome, was used to interpret the variants and assign an ACMG category.^9^ Missense variants were seen most commonly, with frameshift, deletion, duplication, and splice site variants also reported. All patients with variants of uncertain significance had a clinical diagnosis of GSDIb.

**TABLE 1:** Genotype of cohort of patients with GSDIb in England

Nucleotide change	Amino acid change	Variant type	Allele frequency	ACMG category	Previously reported
c.92_94delTCT	p.Phe31del	Deletion	12/62 (19.4%)	Likely pathogenic	Yes
c.1042_1043delCT	p.Leu348fs	Frameshift	8/62 (13%)	Pathogenic	Yes
c.936dupA	p.Val313SerfsX13	Frameshift	8/62 (13%)	Pathogenic	No
c.1105_1106insA	p.Val369AspfsX33	Frameshift	6/62 (9.7%)	Pathogenic	No
c.1243C > T	p.Arg415Ter	Missense	4/62 (6.5%)	Pathogenic	Yes
c.1123 + 3_1123 + 6del	?Truncated protein	Splicing	2/62 (3.2%)	Uncertain significance	No
c.169_175del	p.Ser57fs	Frameshift	2/62 (3.2%)	Pathogenic	Yes
c.55G > A	p.Gly19Arg	Missense	2/62 (3.2%)	Uncertain significance	No
c.1123 + 2dup	?Truncated protein	Splicing	2/62 (3.2%)	Likely pathogenic	No
c.359C > T	p.Pro120Leu	Missense	2/62 (3.2%)	Uncertain significance	No
c.352 T > C	p.Trp118Arg	Missense	2/62 (3.2%)	Pathogenic	Yes
c.742delC	p.Q248fs	Frameshift	1/62 (1.6%)	Pathogenic	Yes
c.1123 + 1G > T	?Truncated protein	Splicing	1/62 (1.6%)	Pathogenic	No
1A > G	?Truncated protein	Missense	1/62 (1.6%)	Pathogenic	Yes
c.81 T > A	p.Asn27Lys	Missense	1/62 (1.6%)	Likely pathogenic	Yes
c.1015G > A	p.Gly339Cys	Missense	1/62 (1.6%)	Likely pathogenic	Yes
c.1015G > T	p.Gly339Cys	Missense	1/62 (1.6%)	Likely pathogenic	Yes
c.285del	p.Trp96fs	Frameshift	1/62 (1.6%)	Pathogenic	No
c.1155C > T	p.Ser385Arg	Missense	1/62 (1.6%)	Uncertain significance	No
c.381 + 5G > C	?Truncated protein	Missense	1/62 (1.6%)	Uncertain significance	No
c.214G > A	p.Asp72Asn	Missense	1/62 (1.6%)	Uncertain significance	No
c.514insG	p.Ser130fs	Frameshift	1/62 (1.6%)	Likely pathogenic	No
c.923_934dup12	p.Met308_Met311dup	Duplication	1/62 (1.6%)	Likely pathogenic	No
c.1175del	p.Ser392fs	Frameshift	1/62 (1.6%)	Likely pathogenic	No

Abbreviation: ACMG, American College of Medical Genetics and Genomics.

### 3.9 Hematopoietic stem cell transplantation

Two patients in our cohort were treated with hematopoietic stem cell transplantation (HSCT). Both were performed in early childhood primarily for neutropaenia and recurrent infections. Both patients are treated with prophylactic antibiotics for post‐HSCT related lung disease and only one has had normalization of their neutrophil count. One patient has been able to extend their fasting tolerance from 4‐5 hourly to 6‐7 hours overnight, and the fasting tolerance of the other patient has not been formally tested following HSCT.

### 3.10 Liver transplantation

Liver transplantation was performed in nine patients with two patients transplanted in adulthood. The two adult patients are both deceased, one due to complications in the immediate post‐operative period and the other as a result of a severe septic episode several years after transplant. Examination of the explanted livers revealed no adenomas and no evidence of cirrhosis. Evidence of mild‐to‐moderate fibrosis was observed in five explanted livers. The pediatric patients received orthotopic liver transplantation at a median age of 3.75 years (range 2.1‐8.6 years) and the most commonly cited reason for transplant was short fasting tolerance and poor quality of life (QoL). All pediatric patients are now able to fast for at least 12 hours overnight and their families anecdotally report a vast improvement in QoL.

**FIGURE 3:**
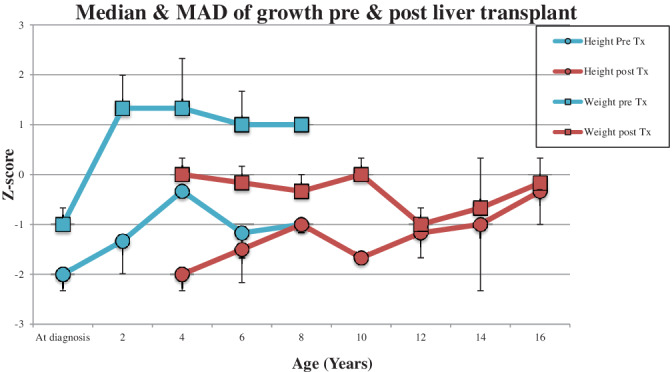
Median and median absolute deviation (MAD) of Z‐scores for growth pre and post liver transplant

Liver transplantation did not improve neutrophil count, with the median neutrophil count persistently remaining below the normal range for all children. Post‐transplant complications include chronic lung disease in one patient, and episodes of rejection in two patients, which have been successfully treated. Another patient has early fibrosis in their graft, which is being monitored. Figure 3 shows the median and MAD for growth in the pediatric patients pre‐ and post‐transplant with a rapid stabilization of weight and a gradual increase in height over time. No statistically significant differences were seen due to low numbers and varied results.

## 4 DISCUSSION

This is the first study of the cohort of GSDIb patients in England to be reported. Our results confirm that even with good biochemical control, growth is very difficult to manage, with the Z‐score for height remaining persistently below zero across childhood and an elevated BMI reported in adulthood. The increase in height Z‐score in Figure 1 between ages 14 and 16 years we feel is due to a sampling bias, with minimal data available for this age range. Short stature is a hallmark feature of GSDI; the etiology of which is poorly understood but which is postulated to be due to a combination of lactic acidosis and disruption to the growth hormone‐insulin‐like growth factor axis. ^10^, ^11^ Current guidelines do not recommend treatment with growth hormone. ^2^, ^5^ Catch‐up growth was seen in the pediatric patients post liver transplant, which is consistent with previous reports. ^12^, ^13^, ^14^

Guidelines on the dietary management of GSDIb are published together with GSDIa, and fasting tolerance is expected to increase with age as glucose requirements decrease.^5^ However, our findings do not completely align with this, with some patients on overnight enteral feeds and 90‐minute daytime feeds in adulthood. This could signify a difference in outcomes between patients with GSDIa and GSDIb. The use of UCCS has undeniably improved metabolic control in patients with GSDI; however, the intolerance to UCCS in patients with GSDIb may be a possible explanation for fasting tolerance not improving with age. Interestingly, two patients appear to have an attenuated form of the disease and both were not diagnosed until adulthood. One has not had their fasting tolerance formally tested and the other is able to fast for 10 hours overnight. In Figure 2, a decrease in fasting tolerance was seen in our cohort between ages 16 and 18 years. We hypothesize that this may be partly due to sampling bias, with minimal data available for the 16 years age group, and might also be due to a loss of metabolic control as patients transition from pediatric to adult services. The dietary management of patients with GSDIb in England involves no formal restriction of sucrose, lactose, or fructose; however, most centers advise patients to avoid simple sugars such as fruit juices.

Renal disease was seen in several of our adult patients and we believe that the albumin/creatinine ratio is a more sensitive marker of early onset renal disease and should be routinely screened for, along with screening for tubulopathy and nephromegaly.^2^ One of our patients required renal replacement therapy and sadly died from post‐operative complications after having a combined liver and kidney transplant. The pathophysiology behind chronic kidney disease in GSDI is believed to be similar to that which is observed in patients with diabetes. ^15^ With the increased life expectancy seen in patients with GSDI we can expect to see a lot more renal complications arise, which may include renal malignancy. ^16^ None of our pediatric liver transplant cohort has had renal complications to date, and it is unclear whether liver transplant conveys a protective factor for renal function in patients with GSDIb. ^13^, ^14^

Gastrointestinal complaints were common in our cohort, with several having either a diagnosis or clinical signs and symptoms suggestive of IBD. This directly correlates with neutropaenia and neutrophil dysfunction and indeed GCSF seems to help ameliorate symptoms. ^17^ Data on dental and periodontal manifestations were not collected; however, anecdotal reports of an increased incidence of tooth decay and gum abscesses were described, in keeping with frequent mouth ulcers. Given the high incidence of complications in those who underwent gastrostomy insertion, we cannot recommend it in this condition. None of our cohort was found to have hepatic adenomas and we would therefore suggest relaxing the frequency of abdominal ultrasound in pediatric patients prior to adolescence to once every 24 months.

Our limited findings on bone and endocrine health are consistent with previous reports, and we would support routine screening as per current guidelines. ^2^, ^5^

The most common variant in our cohort was the c.92_94delTCT variant, resulting in a deletion of phenylalanine and predicted to be likely pathogenic. ^9^ This variant was seen only in patients who had Pakistani and South‐Asian ethnicity. This variant was only reported for the first time in 2019 in a cohort of Brazilian patients.^18^ The second most common variant in our population is the c.1042_1043delCT pathogenic variant causing a frameshift and seen commonly in around 32% of European and German populations and in 33% of the Korean GSDIb population.^1^, ^19^, ^20^, ^21^

Our third and fourth most frequent variants, c.936dupA and c.1105_1106insA, have not been described outside of this cohort.^22^ Both result in a frameshift and are predicted to be pathogenic.^9^ The c.1243C > T variant has been previously reported and is quite close to the C‐terminus so therefore may have a less deleterious effect on protein function.^23^ This variant was seen in two siblings with an attenuated form of GSDIb. In addition to this, we describe 12 novel alleles, of which 6 are predicted to be either pathogenic or likely pathogenic and the remainder are of uncertain significance. ^9^

Two patients in our cohort have had HSCT with only one showing improvement in their neutrophil count. Only one has had definite improvement in their fasting tolerance, albeit this is mild, and may be a reflection of an improved fasting tolerance with age. HSCT has been uncommonly performed for GSDIb and we would not recommend HSCT as a treatment option. ^24^, ^25^ Liver transplantation is recommended for treatment of patients with multiple adenomas, malignant lesions and in extreme cases of growth retardation or poor metabolic control. Liver transplantation did not cause resolution of neutropaenia or recurrent infections in our cohort, which is consistent with previous studies.^12^, ^13^, ^14^ There is no doubt that GSDI has a severe impact on QoL, particularly regarding social functioning and more so in patients with GSDIb.^26^, ^27^ It would be beneficial to extend this study to formally explore the QoL of our cohort and to examine differences between patients who have had liver transplant and those who have not. We believe QoL should be routinely assessed in patients with GSDIb and that poor QoL could be a valid indication for liver transplantation.

Our cohort was anemic and neutropaenic throughout life. The causes of anemia in GSDIb are multifactorial, although it has been proposed that the chronic gastrointestinal inflammation causes up‐regulation of hepcidin expression in the liver, which in turn causes abnormal iron absorption and deficiency. ^2^ The management of neutropaenia in GSDIb may well improve significantly in the future with the more widespread use of SGLT2 inhibitors. The knock‐on effects from this should hopefully see an improvement in anemia, infections, gastrointestinal symptoms and overall QoL. Improved wound healing may also allow for gastrostomy insertion. These drugs have already been commenced on a number of GSDIb patients across the United Kingdom and therefore this natural history study will be important in order to reflect on the GSDIb phenotype in England prior to treatment.

## 5 CONCLUSION

Patients with GSDIb in England have a severe disease with a short fasting tolerance that persists for most of early childhood and does not tend to improve as well over time in comparison with GSDIa. Neutropaenia and recurrent infections persist throughout life, and gastrointestinal symptoms are commonly reported. Liver transplantation does not correct neutropaenia but may be a valid treatment option for poor quality of life.

## CONFLICTS OF INTEREST

Rebecca Halligan, Fiona White, Bernd Schwahn, Karolina Stepien, Nazreen Kamarus Jaman, Mel McSweeney, Steve Kitchen, Joanna Gribben, Charlotte Dawson, Katherine Lewis, David Cregeen, Helen Mundy and Saikat Santra declare that they have no conflict of interest.

## AUTHOR CONTRIBUTIONS

R. Halligan: study design, data collection at two sites, analyses, creation of graphs and figures, writing all drafts. F. J. White: contribution of data. B. Schwahn: contribution of data. K. M. Stepien: contribution of data. N. Kamarus Jaman: contribution of data. M. McSweeney: contribution of diet data. S. Kitchen: contribution of diet data. J. Gribben: contribution of diet data. C. Dawson: contribution of data. K. Lewis: contribution of data. D. Cregeen: contribution to genotype interpretation. H. Mundy: analyses, strategic direction. S. Santra: study design, analyses, strategic direction, revision of drafts.

## COMPLIANCE WITH ETHICS GUIDELINES

All procedures followed were in accordance with the ethical standards of the responsible committee on human experimentation (institutional and national) and with the Helsinki Declaration of 1975, as revised in 2000 (5). Informed consent was obtained from all patients for being included in the study.

## INFORMED CONSENT

This article does not contain any studies on animal subjects performed by any of the authors.
